# State-of-the-Art Pediatric Coronary Artery Bypass Surgery: a Literature Review

**DOI:** 10.21470/1678-9741-2019-0366

**Published:** 2020

**Authors:** Roman Komarov, Alisher Ismailbaev, Vagi Chragyan, Bakytbek Kadyraliev, Michel Pompeu B. O. Sá, Arjang Ruhparwar, Alexander Weymann, Konstantin Zhigalov

**Affiliations:** 1 Department of Cardiovascular Surgery, I.M. Sechenov University Hospital, First Moscow State Medical University, Moscow, Russia.; 2 Department of Cardiovascular Surgery, S.G. Sukhanov Federal Center of Cardiovascular Surgery, E.A. Vagner Perm State Medical University, Perm, Russia.; 3 Department of Cardiovascular Surgery, Pronto Socorro Cardiológico de Pernambuco - PROCAPE, Recife, PE, Brazil.; 4 Department of Thoracic and Cardiovascular Surgery, West German Heart and Vascular Center Essen, University Hospital of Essen, University Duisburg-Essen, Essen, Germany.

**Keywords:** Mammary Arteries, Coronary Vessels, Mucocutaneous Lymph Node Syndrome, Arterial Switch Operation, Pulmonary Artery, Transposition of Great Vessels, Coronary Artery Bypass, Myocardial Revascularization, Heart Defects, Congenital, Iatrogenic Disease

## Abstract

**Objective:**

To examine the results of various myocardial revascularization techniques in pediatric patients to better understand the strategies for surgical treatment of coronary artery pathologies.

**Methods:**

We analyzed 61 publications dedicated to the indications, methods, and results of coronary bypass surgery in children. Due to the small size of this cohort, case reports are also included in our review.

**Results:**

The main indications for coronary bypass grafting in children are Kawasaki disease, myocardial revascularization as a necessary procedure during the congenital cardiac surgery, to manage intraoperative iatrogenic damage to coronary arteries, and homozygous familial hypercholesterolemia. The use of internal thoracic arteries as conduits for coronary bypass grafting in children with Kawasaki disease showed significantly better results in long-term functionality compared to autovenous conduits (87% and 44%, respectively, *P*<0.001). Acute and late coronary events after arterial switch operation for the transposition of the great arteries, anomalous origin of the left coronary artery from the pulmonary artery, and left main coronary artery atresia are the main congenital heart diseases where surgical correction involves interventions on the coronary arteries.

**Conclusion:**

The internal thoracic artery is a reliable and durable conduit that demonstrates proven growth potential in children.

**Table:** 

Abbreviations, acronyms & symbols				
ADA	= Anterior descending artery		KD	= Kawasaki disease
ALCAPA	= Anomalous origin of the left coronary artery from the pulmonary artery		LAD	= Left anterior descending artery
BWGS	= Bland-White-Garland syndrome		LCA	= Left coronary artery
CABG	= Coronary artery bypass grafting		LITA	= Left internal thoracic artery
CAD	= Coronary artery disease		LVEF	= Left ventricular ejection fraction
CHD	= Congenital heart disease		PCABG	= Pediatric coronary artery bypass grafting
CI	= Confidence interval		PCI	= Percutaneous coronary intervention
FFR	= Fractional flow reserve		RCA	= Right coronary artery
FU	= Follow-up		RITA	= Right internal thoracic artery
ITA	= Internal thoracic artery		TGA	= Transposition of the great arteries

## INTRODUCTION

Over the past few decades, there has been almost no controversy with respect to different approaches in myocardial revascularization in adult patients with coronary artery disease (CAD). Hence, clear criteria have been developed for choosing a particular technique of myocardial revascularization - in particular, percutaneous interventions, coronary artery bypass grafting (CABG), or hybrid techniques, depending on the severity of myocardial ischemia (acute coronary syndrome), the degree and morphology of coronary artery lesions, and the presence of CAD complications and comorbid pathology^[1]^. In addition, both early and long-term outcomes of each intervention have been thoroughly studied in adult patients with CAD^[2]^.

**Fig. 1:**
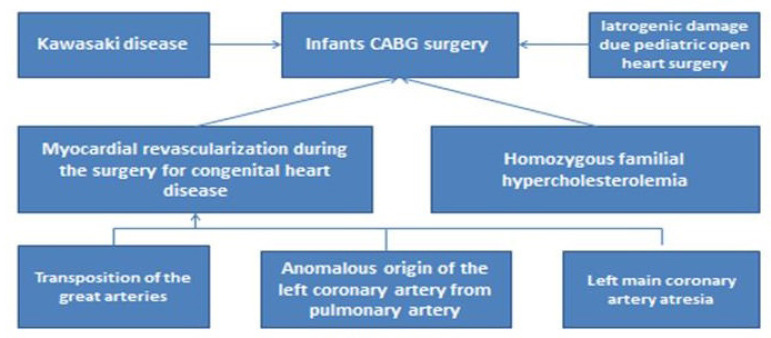
Main indications for pediatric coronary artery bypass grafting (CABG) surgery. LCA=left coronary artery

Nowadays, the rapid development of endovascular, surgical, and hybrid techniques has led to the introduction of various methods of myocardial revascularization in pediatric patients. Indications for coronary artery intervention in children are quite broad and include the following spectrum of pathologies^[3]^ (Figure 1).

1. Kawasaki disease (KD);
2. Myocardial revascularization as a necessary procedure during the surgery for congenital heart disease (CHD);
3. Iatrogenic damage to coronary arteries during the surgery for CHD;
4. Homozygous familial hypercholesterolemia.

However, given the rapid growth of children and their increasing physical activity over the years, the strategy of myocardial revascularization should differ from that in adult patients, which requires careful analysis of both early and long-term outcomes of a particular revascularization technique for various coronary artery pathologies^[4]^. To better understand the strategies for surgical treatment of coronary artery pathologies in children, it is necessary to examine the results of various revascularization techniques in the context of the indications mentioned above. Table 1 represents the best evidence publications regarding myocardial revascularization in pediatric patients.

**Table 1:** Best evidence publications for pediatric coronary artery bypass grafting (PCABG).

Author (date), journal, country and study type	Patient group	Key results	Conclusions
Kitamura et al.^[37]^ (2009), Circulation, Japan. Single-center retrospective study.	114 patients who underwent PCABG for KD using ITA and saphenous vein grafts. Median age - 10 years (1 to 19 years). Median FU time - 19 years (up to 25 years)	No in-hospital mortality. 25-year survival rate - 95%. Cardiac event-free rates at 20 and 25 years were 67% and 60% (95% CI, 46 to 72), respectively. The 20-year graft patency rate was 87% (95% CI, 78 to 93) for internal thoracic artery grafts (n=154) and 44% (95% CI, 26 to 61) for saphenous vein grafts (n=30) (P<0.001)	PCABG for KD showed excellent long-term outcomes. ITA was the most favorable graft for pediatric patients.
Vida et al.^[12]^ (2013), Ann. Thorac. Surg., Italy. Multi-center retrospective study.	80 patients from 13 centers who underwent PCABG and other coronary artery procedures between 1973 and 2011. KD patients were excluded. Median age - 2.3 years (2 days to 16.9 years). Median FU time - 7.6 years (range, 0.9 to 23 years).	In-hospital mortality - 15% (12 patients). Three late cardiac deaths after a median FU time of 4 years (range, 9 months to 8.8 years). Reintervention rate during FU - 7.5% (6 patients).	PCABG is a suitable surgical option in pediatric patients with impaired myocardial perfusion, which increases mid-term survival. Life-long FU needed to prevent and treat any further coronary/myocardial complications.
Legendre et al.^[13]^ (2010), J. Thorac. Cardiovasc. Surg., France. Single-center retrospective study.	18 patients who underwent PCABG using LITA and/or RITA between 1988 and 2007. Median age - 4 months (3 days to 35 months). Median FU time - 41 months (1 to 176 months)	No in-hospital mortality. During FU, 1 graft was occluded and 2 needed a percutaneous intervention. Two patients died 3.5 and 4.6 months post-PCABG, respectively.	PCABG should be considered as a possible alternative for coronary revascularization. This procedure remains a technical challenge and requires careful FU.
Mavroudis et al.^[28]^ (1999), Ann. Thorac. Surg., United States of America. Single-center retrospective study.	16 patients who underwent PCABG using LITA and/or RITA for KD, congenital lesions, post arterial switch, and other iatrogenic obstructions between 1987 and 1998. Mean age - 6.1 years (2 months to18 years). FU range - 2 months to 11 years.	Overall survival - 93.8% (15 patients). All bypass grafts in surviving patients were patent during FU.	PCABG using ITA can be successfully performed in infants and children for expanding elective and life-saving indications with excellent results.

CI=confidence interval; FU=follow-up; ITA=internal thoracic artery; KD=Kawasaki disease; LITA=left internal thoracic artery; RITA=right internal thoracic artery

## INDICATIONS FOR CORONARY ARTERY INTERVENTION IN CHILDREN

### Kawasaki Disease

KD, or mucocutaneous lymph node syndrome, is an acute systemic disease characterized by the development of vasculitis with frequent coronary and other visceral artery lesions. It is the main cause of acquired heart disease in children, being more common than acute rheumatic fever in the last few years^[5]^. World literature data indicates the endemic nature of this disease - Japan is the recognized “leader” in the prevalence of KD (100-110 per 100,000 children). The approximate prevalence is about 4.4 cases per 100,000 children. In the article of Shirinskaya OG. et al.^[5]^, 90 patients with KD were observed from 2004 to 2010^[5,6]^. The most dangerous complication of KD is cardiac involvement (12.5-50%) with pathognomonic lesions in coronary arteries - coronary aneurysms occur in 20-40% of cases^[7]^. According to the recommendations of the American Heart Association (published in 2004), coronary aneurysms are divided into:

- Small (inner diameter < 5 mm);
- Medium (5-8 mm);
- Giant (> 8 mm)^[8]^.

**Fig. 2:**
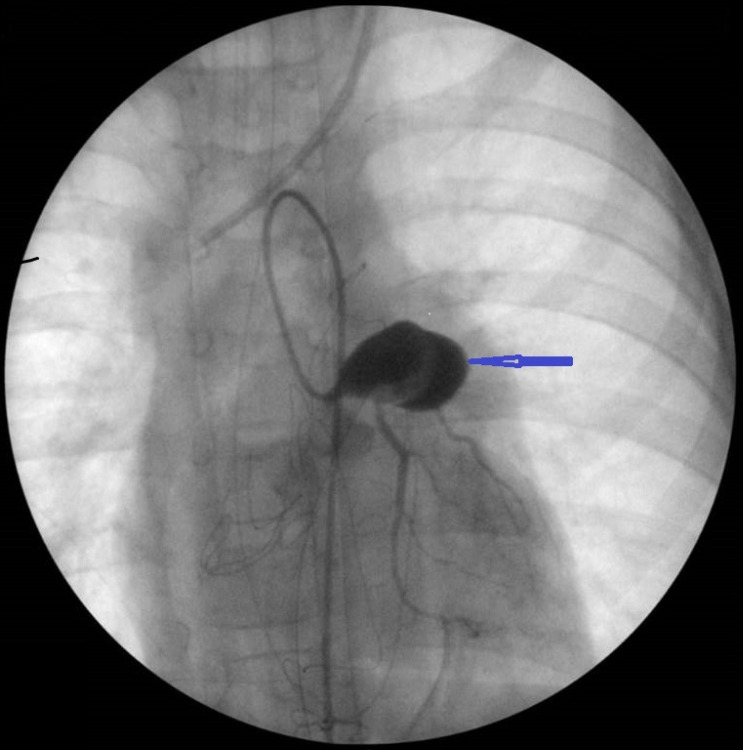
Giant aneurysm of the left coronary artery trunk in a 7-year-old child with Kawasaki disease.

Despite the data on complete regression of coronary aneurysms in 50-60% of cases within 1-2 years, the mortality rate in children without medical therapy is 1-2%, and up to 0.08% with immunoglobulin treatment^[9]^. In addition, Kato et al.^[10]^ reported that significant coronary artery lesions persist in most children even after the convalescence^[10]^. In this cohort of patients, mortality rates were 22.0%, 62.5%, and 83.3% after the first, second, and third episodes of myocardial infarction respectively, thereby demonstrating the need for more effective treatment, including a surgical approach^[11]^. Some authors classify coronary artery lesions as aneurysms with stenosis or without stenosis^[3]^. According to most authors, obstructive lesions of coronary arteries almost always include areas of entry or exit from aneurysms, localized more often in the proximal parts of the arteries, especially in the main left coronary artery (LCA) or left anterior descending artery (LAD)^[3]^ (Figure 2). On the other hand, lesions of the right coronary artery (RCA) are often localized in more distal parts: proximal, and sometimes, distal to bifurcation^[12]^.

### Myocardial Revascularization as a Necessary Procedure During the Surgery for CHD

There are *three* CHDs where the surgical correction involves interventions on the coronary arteries^[13]^: 1) acute and late coronary events after arterial switch operation for the transposition of the great arteries (TGA); 2) anomalous origin of the LCA from the pulmonary artery (ALCAPA); and 3) left main coronary artery atresia.

Problems with the transfer of coronary arteries during arterial switch operation for TGA or Taussig-Bing anomaly are the main cause of early postoperative mortality, which occurs in 7-8% of newborns and children older than one year, whereas the frequency of late coronary stenosis or occlusion is 11.3%^[14]^. Tsuda T. et al. retrospectively examined the morphology of coronary arteries in 40 patients in the long term after the switch operation^[14]^. Significant late changes in coronary arteries were found in seven children. There was one case of sudden cardiac death at the age of 3.8 years^[14]^. The critical stenosis of main LCA was revealed during autopsy. Another child at the age of 9.6 years developed severe ventricular arrhythmias in the presence of similar changes in the main LCA, despite the negative treadmill test. In another patient, moderate stenosis of LCA was detected; however, according to the treadmill test and myocardial scintigraphy, no ischemia was detected. There was a case of ST-T depression during physical activity and impaired myocardial perfusion, according to the results of positron emission tomography scan, with complete occlusion of main LCA and the formation of collateral vessels. In three other children, complete proximal occlusion of all major coronary arteries with collateral blood flow was found^[15]^. Ou P. et al.^[15]^ examined the mechanisms of coronary complications after the switch operation using three-dimensional multidetector computed tomography. The anterior repositioning of the LCA (between 12 and 1 hours on the neoaorta) predisposes to its tangential course and stenosis. All circular lesions were detected in Type B and D malformations, according to Yakub, where the initially long retroaortic artery was stretched during its reimplantation into the neoaorta. Stenosis of the RCA was found in cases where the reimplantation site was located high above the right coronary sinus with potential compression from the bifurcation of the pulmonary trunk^[16]^.

Bergoënd E. et al.^[16]^ published their treatment experience of 25 patients (mean age 5.3 years) who underwent myocardial revascularization after a previous arterial switch operation. In eight children (mean age 8.0 years), CABG with internal thoracic artery grafts were used, while 17 patients underwent arterioplasty of the main LCA. One patient died four days after arterioplasty due to cardiogenic shock. Three months after CABG, one child died from an unknown cause and two patients died from graft dysfunction and occlusion of anastomosis. Remaining patients in the average follow-up period of 3.4 years after arterioplasty and 4.4 years after CABG were alive. Two children, 2.6 and 5.7 years after the arterioplasty, developed LAD restenosis, which required CABG. Thus, the long-term survival after arterioplasty and CABG was respectively 72% and 63%^[16]^. Prifti E. et al.^[17]^ presented the experience of myocardial revascularization in two children after the switch operation. One child, operated at the age of three days for TGA (Type “A”), with a fairly simple reimplantation of the coronary arteries into the neoaorta, was admitted three months later with acute coronary syndrome. Emergency coronary angiography detected stenosis of LAD (75%) and proximal RCA occlusion. The child underwent emergency CABG using both internal thoracic arteries. The early postoperative period was uneventful, and the control coronary angiography after 12 months showed a good patency of both grafts and anastomoses^[17]^. The second newborn with TGA (Type “B”) and Rashkind procedure on the 6^th^ day of life, was admitted two weeks after arterial switch operation in Le Compte modification with symptoms of tachypnea. Electrocardiogram revealed anterolateral myocardial infarction, while emergency coronary angiography detected subtotal stenosis of the only coronary trunk, occlusion of LCA, moderate stenosis of the proximal third of the left circumflex artery, and unsatisfactory diameter of left internal thoracic artery. Arterioplasty of the common coronary trunk, left and right coronary arteries with an autopericardial patch in the form of trousers fixed from the aortotomy incision to the distal parts of the arteries, was performed. The postoperative period was uneventful. After nine months, coronary angiography showed good patency of both coronary arteries^[17]^.

According to most authors, reimplantation of the LCA into the aorta at the early age is the “gold” standard for the treatment of patients with Bland-White-Garland syndrome (BWGS), also well-known as ALCAPA syndrome. This procedure allows full restoration of myocardial function during the first year after surgery^[18,19]^. According to various reports, the survival of patients with this CHD is directly correlated with the degree of collateral coronary blood flow development: most patients die in infancy, the so-called “infantile type”, while patients with well-developed collateral blood flow, the so-called “adult type”, in rare cases, live up to 50-60 years^[20]^. The most important and most common complications of BWGS include ischemic mitral regurgitation and severe ventricular arrhythmias, which often develop in elderly patients^[21]^. Given the complexity of the anatomy, as well as the technical aspects of reimplantation of LCA into the aorta, some authors described cases (more often in adults than in children) where CABG was necessary^[22]^. A large work published by the Texas Heart Institute in 2002 and dedicated to 37 years’ experience of surgical treatment of coronary artery anomalies described the treatment results of 16 children who underwent ligation of LCA with anomalous origin from pulmonary artery with subsequent CABG using great saphenous vein and left internal thoracic artery^[23]^. The authors showed no differences in the early patency of anastomoses using great saphenous vein or left internal thoracic artery; however, the late venous graft patency was only 70%, which indicated the use of internal thoracic artery as the preferred graft. As noted above, reimplantation of LCA into the aorta is the treatment of choice for this CHD and is technically possible in most patients in infancy. CABG after ligation of the main LCA can be a good alternative when there is a high risk of artery tension because of a large distance between the origin of the artery and the aorta^[18]^.

Left main coronary artery atresia is a rare congenital defect with a nonspecific and diverse presentation, ischemic complications in the neonatal period, including early ventricular dysfunction and mitral regurgitation^[24]^. In a literature review, Musiani et al.^[25]^ presented data of 28 patients with left main coronary artery atresia: 15 children and 13 adults. Pediatric patients in this review were of different ages. Their symptoms included shortness of breath, loss of consciousness, myocardial infarction, ventricular tachycardia, and sudden death. Congenital heart defects identified in five children were supravalvular aortic stenosis (n=2), stenosis of the ostium of the RCA (n=1), ventricular septal defect (n=1), and pulmonary valve stenosis (n=1)^[25]^. The authors noted that in adult patients, CABG is definitely the procedure of choice, since the use of left internal thoracic artery demonstrated excellent long-term patency and greater physiological adaptability to various blood flow patterns. However, in pediatric patients, long-term CABG results may be doubtful, especially when using great saphenous vein grafts^[13]^. In general, there are reports of only 12 attempts at myocardial revascularization in children with left main coronary artery atresia^[26]^. However, in the case of atresia of long LCA segments in pediatric patients, CABG using left internal thoracic artery is the only option^[27]^.

### Iatrogenic Damage to the Coronary Arteries During the Surgery for CHD

Many authors consider iatrogenic damage to the coronary arteries as the reason to perform myocardial revascularization in pediatric practice^[3,28]^. Thus, in a multicenter study, Vida V. et al.^[12]^ showed data of 80 children who underwent various coronary interventions in several European centers over the past 30 years. It should be noted that the study included patients who underwent “open” heart surgery for CHD, while the cause of coronary events in 43 (53.8%) children was iatrogenic damage to the coronary arteries during the main surgical procedure, which was associated with the anomalous topography of the coronary vessels in 27 of 43 patients (62.8%; =0.028). The following grafts were used for CABG: left internal thoracic artery (70%), right internal thoracic artery (17%), and great saphenous vein in 13% of cases. Hospital mortality was 18%, and graft dysfunction was detected in only one case. The main causes of high mortality were left or right ventricular infarction and complications associated with extracorporeal membrane oxygenation.

### Homozygous Familial Hypercholesterolemia

Familial hypercholesterolemia is one of the most common inherited cardiovascular diseases, affecting up to 1/250 people worldwide and about 1.3 million people in the United States of America^[29]^. A homozygous type is rare and causes tendon xanthomas and CAD in the first years of life^[30]^. The cause is the mutation of the low-density lipoprotein gene^[31]^. It is the homozygous type that leads to CAD in the first few years of life, which in some cases determines the need for direct myocardial revascularization^[32]^. In addition, the natural course of the disease is characterized by frequent damage to the aortic valve with critical stenosis formation, and the only radical method of treatment is liver transplantation as soon as possible after diagnosis^[30,33]^.

Despite the good results of endovascular revascularization in the adult patients with familial hypercholesterolemia, especially using bioabsorbable stents, most authors are unanimous in the unacceptability of this strategy in pediatric practice and the preference for CABG^[34,35]^. Literature review indicates the obligatory use of left or both internal thoracic arteries, while venous grafts should be used only in the presence of a multivascular lesion for complete myocardial revascularization. Some reports described successful staged treatment of homozygous hypercholesterolemia in children with CABG followed by liver transplantation^[31]^.

## RESULTS OF SURGICAL TREATMENT OF KAWASAKI DISEASE WITH CORONARY ARTERY LESIONS

### Coronary Artery Bypass Grafting

The first case of CABG in a four-year-old boy with KD and myocardial infarction with complete occlusion of both LAD and RCA, as well as a reduced left ventricular ejection fraction (LVEF) (45%), was published by Kitamura S. et al.^[36]^ in 1976. The section of the great saphenous vein of the left thigh was used as conduits, and postoperative angiograms showed excellent patency of the anastomoses with an improvement in LVEF in the early postoperative period up to 61%. Unfortunately, both grafts completely closed within two years; the child survived only because of the presence of intercoronary collateral vessels, but 10 years after CABG, absolute indications for heart transplantation have emerged^[11]^. It should be noted that autovenous conduits were used exclusively in the early stages of the development of pediatric CABG in the treatment of KD; because of unsatisfactory long-term patency, it was decided to abandon the routine use of this graft, especially in children under four years of age^[37]^.

In 1985, Kitamura S. et al.^[38]^ reported for the first time the successful use of both internal thoracic arteries in the treatment of KD. The defining aspect of this surgical approach was the fact that internal thoracic arteries are rarely susceptible to autoimmune damage in KD because of the prevalence of elastic fibers in the vessel wall, in contrast to coronary and peripheral arteries, which consist mainly of muscle fibers^[39]^.

**Fig. 3:**
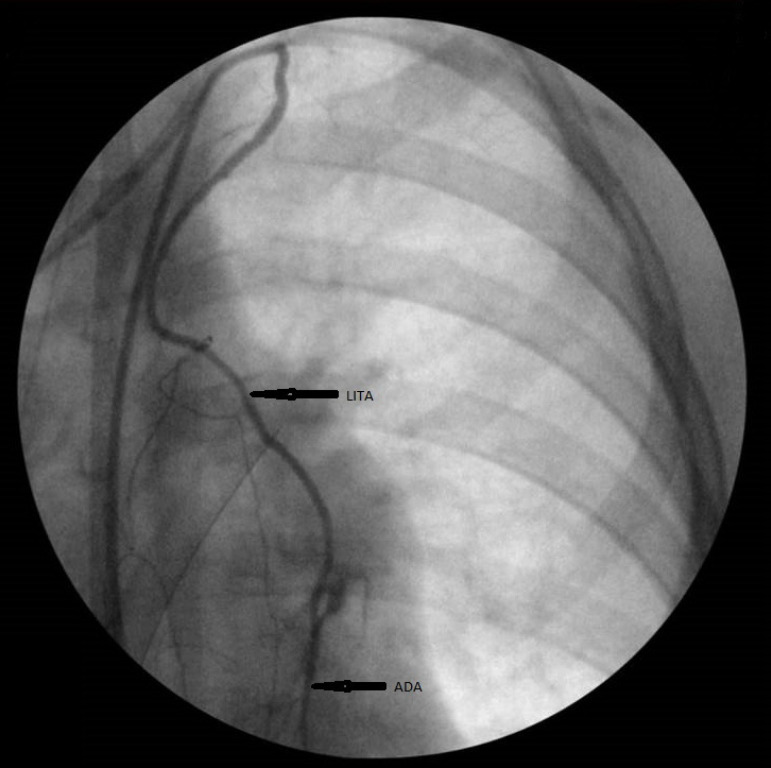
Intraoperative coronary angiogram of anterior descending artery (ADA) grafting using left internal thoracic artery (LITA) in a 7-year-old child with Kawasaki disease.

Important aspects of CABG in children with KD are choosing on-pump or off-pump bypass surgery, as well as monitoring the quality of anastomoses, considering the extremely small diameter of the vessels. The latest published large-scale work by Kitamura S. et al.^[3]^ indicated a clear desire to perform off-pump CABG; however, none of the publications provided exact data on the on-pump conversion rate. Nevertheless, there are some observations that indicate a rather successful use of cardiopulmonary bypass in pediatric practice^[40]^. It is important to keep in mind that internal thoracic arteries are short and thin-walled vessels with a diameter of 1 mm or less, which corresponds to the small size of the chest in children. This requires careful and accurate dissection of the native arteries using surgical optics with a large magnification or a microscope, as well as 9-0, 10-0, or 11-0 threads with a minimal needle size^[41]^. In bypass surgery in adult patients, a surgical microscope is usually not used; however, its use is associated with the high quality of anastomoses in pediatric practice^[42]^. In addition, most publications on CABG in children indicate the routine performance of intraoperative coronary angiogram in all patients, although in adult patients, this is routinely performed only in the Bakulev Scientific Center for Cardiovascular Surgery^[3,43]^ (Figure 3).

### Conduit Response to the Somatic Growth of Children

**Fig. 4:**
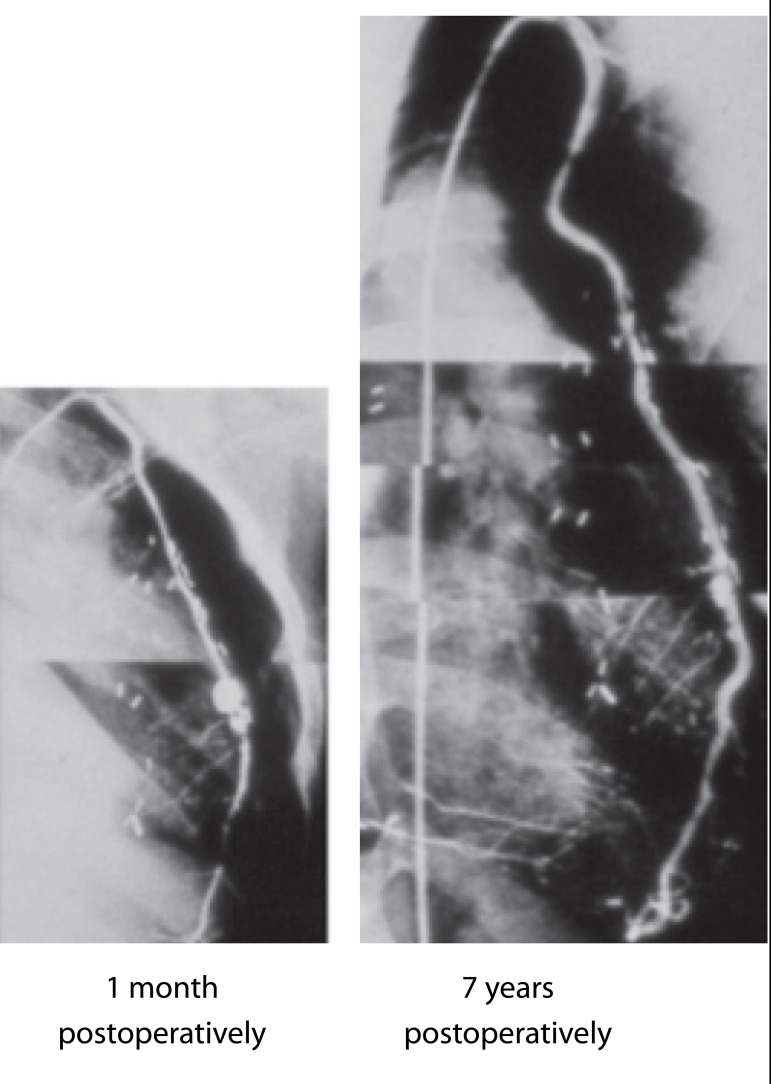
The longitudinal growth of the internal thoracic artery in accordance with the somatic development of the child from 1 month to 7 years after coronary artery bypass grafting. The growth potential is obvious, without any degenerative changes in the graft wall.

Another important, but unresolved, problem remains the response of the conduit to the somatic growth of children. In 1988, Kitamura S. et al., for the first time, published the results of mathematical modeling of the growth potentials of the internal thoracic arteries in the longitudinal and transverse directions in accordance with the somatic growth of the child^[44,45]^ (Figure 4). In addition, the authors demonstrated that the use of both internal thoracic arteries did not lead to any long-term adverse consequences for the development of the chest in children^[37]^. It has been proven that the right and left internal thoracic arteries have exactly the same wall structure and demonstrate similar functionality of anastomoses, identical growth potential, and very low incidence of late degeneration^[7]^.

Autovenous conduits, which had been widely used in the early stages of pediatric coronary surgery development, showed a complete lack of longitudinal growth potential, as well as significantly worse of early and long-term patency compared with internal thoracic arteries, because of rapid intimal hyperplasia and early atherosclerosis, especially in children under 10 years old^[46]^.

### Data on the Long-Term Patency and Morphology of Grafts

As noted above, the use of internal thoracic arteries as grafts for CABG in children with KD showed significantly better results in the long-term functionality compared to autovenous conduits^[12,37]^. In addition, the long-term patency of the internal thoracic arteries did not depend on the age of the patients (under or over 10 years old), and, in the majority of cases, the functionality of the anastomoses did not change within 25 years after the surgery^[47]^. According to some studies, early occlusion of the internal mammary arteries was rarely noted. However, in 20-25% of patients, complete spontaneous recanalization of the grafts occurred within a few years after CABG^[48]^. In turn, occlusion of autovenous conduits (great saphenous vein) occurred both within one year after the surgery and in the long term^[49]^. According to Kitamura S. et al.^[3]^, CABG - using both internal thoracic arteries and performed in childhood - allows the maintenance of blood supply to the heart in adulthood. According to the results of a large study in Japan, the 25-year patency of internal thoracic artery grafts and autovenous conduits was 87% and 44%, respectively (*P*<0.001)^[37]^. At the same time, the long-term functionality of the internal thoracic artery grafts implanted in children younger or older than 10 years did not differ (*P*=0.163), compared with 25% patency of autovenous conduits (*P*<0.004). According to the results of a 30-year-long postoperative observation by Kitamura S., it was concluded that the internal thoracic artery is the conduit of choice in the pediatric coronary surgery, characterized by high patency, growth potential, and metabolic benefits for many decades^[50]^.

### Negative Aspects of Using Internal Thoracic Artery Grafts in Pediatric Practice

The main cause of internal thoracic artery graft dysfunction in children does not differ from that in adults with CAD. The disturbance of patency occurs because of competitive blood flow and high volumetric velocities in native coronary arteries^[51,52]^. Nevertheless, as noted above, in 20-25% of patients with graft dysfunction, complete recanalization was observed at various times after CABG, which is explained by the peculiarities of endothelial function in children, as well as the gradual disappearance of competitive blood flow because of the progression of obstructive lesions in native arteries^[48]^. Owing to the cases of graft dysfunctions, some authors consider measuring fractional flow reserve (FFR) before surgery^[53]^. Ogawa et al.^[53]^ demonstrated that reduced FFR normalizes after successful CABG in patients with KD. Botman et al.^[54]^ studied the importance of two FFR values (> 0.75 or ≤ 0.75) in internal thoracic artery graft dysfunction in adults, and showed that 8.9% of grafts were occluded in patients with significant coronary artery lesions (FFR ≤ 0.75) and 21.4% of grafts with insignificant coronary lesions (FFR > 0.75). However, the authors stated that these differences were not statistically significant, due to the lack of differences in the prevalence of angina pectoris or repeated interventions in any of the groups. The value of measuring FFR in preventing graft dysfunction in children with coronary aneurysms requires further prospective evaluation^[3]^.

### Long-Term Results of CABG in Children with Kawasaki Disease

It has been proven that CABG is a safe procedure for children with severe coronary artery lesions due to KD^[3]^. Mortality in the early postoperative period in the group of more than 100 patients was 0%, and in the next 25 years, it was 5% (95% confidence interval [CI]: 2-12)^[37]^. According to Kitamura S. et al.^[37]^, successful revascularization normalizes cardiac function and coronary blood flow, which is very important for growing children whose physical activity increases with somatic growth. These authors showed that the 30-year survival rate of children with KD and myocardial infarction was 49% (95% CI: 27-71),) compared with 95% (95% CI: 88-98) if myocardial revascularization was performed^[55]^.

The cause of late mortality after CABG was sudden cardiac death, which occurred more often in children with reduced left ventricular function and ventricular tachyarrhythmias^[56]^. Thus, the strategy of rigorous patient monitoring in the early postoperative period (checking functionality of anastomoses, electrophysiological studies, radiofrequency ablation, and implantation of a cardioverter-defibrillator in case of ventricular arrhythmias) directly affects long-term mortality. In the literature, 15 cases of heart transplantation for children with KD, severe decrease in LVEF, and multiple giant coronary aneurysms are described^[57]^.

The main adverse cardiac events after CABG of children with KD are caused by: 1) graft stenosis including the area of anastomoses; 2) graft obstruction, including competitive blood flow; 3) fibroproliferative thickening of intima and atherosclerosis of autovenous conduits; 4) late thrombosis of coronary aneurysms; and 5) post-inflammatory progression of fibro-obstructive lesions in native arteries^[37]^. According to Kitamura S. et al.^[37]^, freedom from adverse cardiac events five, 10, 20, and 25 years after surgery was 87%, 81%, 70%, and 62% (95% CI: 48-74), respectively (Figure 2). Tsuda et al.^[56]^ reported that 30-year freedom from cardiac events in unoperated children with giant aneurysms (≥ 8 mm in diameter) was 36% (95% CI: 28-45, n=245), and 62% (95% CI: 48-74, n=114) in patients after CABG. Despite the rather frequent cardiac events over the 25-year follow-up period, cases with fatal outcomes were rare, which is explained by timely and adequate repeated interventions, including percutaneous coronary interventions (PCI)^[37]^. In addition, since the majority of patients reached their adulthood by the time when additional interventions were required (repeat CABG using either radial and/or gastro-omental artery, or PCI), its outcome was much more successful than in children^[58]^. According to large studies, 84% of operated children could attend sport programs at school, and 16% of them were actively involved in sports^[37]^. Many patients who underwent CABG in childhood due to KD were successfully employed, and several patients had successful pregnancies and childbirths^[56]^.

### Treatment of Coronary Aneurysms Without Stenosis

As noted above, CABG in children with KD and arterial aneurysms may be associated with a risk of graft dysfunction in the presence of competitive blood flow. Treatment of aneurysms without stenosis or occlusion, therefore, is particularly difficult. The main treatment goal is to prevent acute thrombotic obstruction in coronary aneurysms. Several surgical approaches have been described, in addition to anticoagulant and antiplatelet therapy, which mainly include the combination of warfarin and aspirin.

### Ligation of Coronary Aneurysms and Artery Bypass

Some authors insist on the need for aneurysm resection or ligation of the inlet and/or outlet segments in order to avoid their rupture^[59]^. Nevertheless, considering the rarity of aneurysmal rupture, with the exception of the acute inflammatory phase of KD, the practice of native artery ligation with bypass surgery of distal parts is rarely described in the literature^[60]^. Kitamura S. reported a case of RCA aneurysm outlet ligation in combination with CABG using internal thoracic artery grafts. In the early postoperative period, the child developed a complete right bundle branch block and a marked increase in the level of cardiac enzymes^[7]^. The authors explained these consequences by the insufficient volumetric blood flow provided by the internal thoracic artery and recommended avoiding ligation of native coronary arteries, referring to the reversibility of graft dysfunction in the presence of competitive blood flow. The optimal surgical strategy in such situations is CABG using one or both internal thoracic arteries or great saphenous vein, despite the high probability of late graft dysfunction^[7]^.

### Reducing the Size of Coronary Aneurysms

This procedure was first proposed by Abe et al.^[61]^, who excised the non-calcified anterior wall of a giant RCA aneurysm and restored arterial integrity under the control of an appropriately sized probe. As important hypothetical advantages of this procedure, the authors considered a decrease in blood flow turbulence in the affected area, as well as withdrawal of anticoagulant therapy. However, in the long term, all patients developed coronary artery thrombosis.

## CONCLUSION

CABG, as a method of myocardial revascularization in children - in contrast to surgical treatment of CAD in adult patients - is not frequent, but, at the same time, not a fully understood problem. The range of indications for CABG in pediatric practice is quite wide, but it is most often used to treat coronary complications of KD. Direct myocardial revascularization is an integral part of surgical treatment for some CHD, like TGA, BWGS, and coronary atresia. The frequent occurrence of iatrogenic damage to the coronary arteries during the surgery for CHD indicates the need for a thorough preoperative evaluation of children to reveal the anomalies in the topography of coronary arteries. Despite the ongoing discussion about the advantages and disadvantages of different grafts in adult coronary surgery, the position is clear in pediatric practice: the internal thoracic artery is a reliable and durable conduit that demonstrates the proven growth potential in children.

**Table:** 

Authors' roles & responsibilities	
RK	Substantial contributions to the conception or design of the work; or the acquisition, analysis, or interpretation of data for the work; drafting the work or revising it critically for important intellectual content; final approval of the version to be published
AI	Substantial contributions to the conception or design of the work; or the acquisition, analysis, or interpretation of data for the work; drafting the work or revising it critically for important intellectual content; final approval of the version to be published
VC	Substantial contributions to the conception or design of the work; or the acquisition, analysis, or interpretation of data for the work; drafting the work or revising it critically for important intellectual content; final approval of the version to be published
BK	Substantial contributions to the conception or design of the work; or the acquisition, analysis, or interpretation of data for the work; drafting the work or revising it critically for important intellectual content; final approval of the version to be published
MPBOS	Substantial contributions to the conception or design of the work; or the acquisition, analysis, or interpretation of data for the work; drafting the work or revising it critically for important intellectual content; final approval of the version to be published
AR	Substantial contributions to the conception or design of the work; or the acquisition, analysis, or interpretation of data for the work; drafting the work or revising it critically for important intellectual content; final approval of the version to be published
AW	Substantial contributions to the conception or design of the work; or the acquisition, analysis, or interpretation of data for the work; drafting the work or revising it critically for important intellectual content; final approval of the version to be published
KZ	Substantial contributions to the conception or design of the work; or the acquisition, analysis, or interpretation of data for the work; drafting the work or revising it critically for important intellectual content; final approval of the version to be published
